# Determinants of multimorbidity in older adults in Iran: a cross-sectional study using latent class analysis on the Bushehr Elderly Health (BEH) program

**DOI:** 10.1186/s12877-024-04848-y

**Published:** 2024-03-11

**Authors:** Maryam Marzban, Ali Jamshidi, Zahra Khorrami, Marlous Hall, Jonathan A Batty, Akram Farhadi, Mehdi Mahmudpour, Mohamad Gholizade, Iraj Nabipour, Bagher Larijani, Sima Afrashteh

**Affiliations:** 1 Statistical Genetics Lab, QIMR Berghofer Medical Research Institute, QLD Brisbane, Australia; 2 The Persian Gulf Tropical Medicine Research Center, The Persian Gulf Biomedical Sciences Research Institute, Bushehr University of Medical Sciences, Bushehr, Iran; 3 Ophthalmic Research Center, Research Institute for Ophthalmology and Vision Science, Shahid Beheshti University of Medical Sciences, Tehran, Iran; 4 Leeds Institute of Cardiovascular and Metabolic Medicine, University of Leeds, Leeds, UK; 5 Leeds Institute for Data Analytics, University of Leeds, Leeds, UK; 6 The Persian Gulf Marine Biotechnology Research Center, The Persian Gulf Biomedical Sciences Research Institute, Bushehr University of Medical Sciences, Bushehr, Iran; 7 Endocrinology and Metabolism Research Center, Endocrinology and Metabolism Clinical Sciences Institute, Tehran University of Medical Sciences, Tehran, Iran; 8 Department of Biostatistics and Epidemiology, Faculty of Health and Nutrition, Bushehr University of Medical Sciences, Bushehr, Iran

**Keywords:** Latent class analysis, Multi-morbidity, Prevalence, Elderly, Chronic disease

## Abstract

**Background and objectives:**

Multimorbidity, defined as the presence of two or more long-term health conditions in an individual, is one of the most significant challenges facing health systems worldwide. This study aimed to identify determinants of classes of multimorbidity among older adults in Iran.

**Research Design and methods:**

In a cross-sectional sample of older adults (aged ≥ 60 years) from the second stage of the Bushehr Elderly Health (BEH) program in southern Iran, latent class analysis (LCA) was used to identify patterns of multimorbidity. Multinomial logistic regression was conducted to investigate factors associated with each multimorbidity class, including age, gender, education, household income, physical activity, smoking status, and polypharmacy.

**Results:**

In 2,426 study participants (mean age 69 years, 52% female), the overall prevalence of multimorbidity was 80.2%. Among those with multimorbidity, 3 latent classes were identified. These comprised: class 1, individuals with a low burden of multisystem disease (56.9%); class 2, individuals with predominantly cardiovascular-metabolic disorders (25.8%) and class 3, individuals with predominantly cognitive and metabolic disorders (17.1%). Compared with men, women were more likely to belong to class 2 (odds ratio [OR] 1.96, 95% confidence interval [CI] 1.52–2.54) and class 3 (OR 4.52, 95% CI 3.22–6.35). Polypharmacy was associated with membership class 2 (OR 3.52, 95% CI: 2.65–4.68) and class 3 (OR 1.84, 95% CI 1.28–2.63). Smoking was associated with membership in class 3 (OR 1.44, 95% CI 1.01–2.08). Individuals with higher education levels (59%) and higher levels of physical activity (39%) were less likely to belong to class 3 (OR 0.41; 95% CI: 0.28–0.62) and to class 2 (OR 0.61; 95% CI: 0.38–0.97), respectively. Those at older age were less likely to belong to class 2 (OR 0.95).

**Discussion and implications:**

A large proportion of older adults in Iran have multimorbidity. Female sex, polypharmacy, sedentary lifestyle, and poor education levels were associated with cardiovascular-metabolic multimorbidity and cognitive and metabolic multimorbidity. A greater understanding of the determinants of multimorbidity may lead to strategies to prevent its development.

## Introduction

Multimorbidity is defined as the simultaneous presence of two or more chronic diseases in an individual [1]. The prevalence of multimorbidity has been on the rise in recent years, resulting in significant impacts on affected individuals, their families, healthcare systems, and society [2]. Population-based cohort studies of older adults report a prevalence of multimorbidity ranging from 19 to 63%, depending on the definitions used and the population studied [3–5]. According to the Kharameh cohort study, the prevalence of multimorbidity among Iranian adults aged between 40 and 70 has been reported to be 24.4% [6]. However, the causes of this increase in the prevalence of multimorbidity are still debatable, which can vary in each population based on the differences in ethnic, socioeconomic factors, and levels of health, especially in older adults.

By 2050, more than 21% of the world’s population is projected to be over 60 years old, and 80% of this group will live in low- and middle-income countries [7]. Iran has one of the most rapidly aging populations in the world [8]. As a result of improvement in evidence-based public health interventions, improved medical care, and population aging, the life expectancy is prolonging and the prevalence of multimorbidity is increasing [1, 9]. According to reports, there are several factors that may increase an individual’s risk of developing multimorbidity, including advancing age, low socioeconomic status, high body mass index, and smoking [7, 10].

Previous studies have associated multimorbidity with decreased physical function [11], frailty [12], disability, polypharmacy [13], increased usage of healthcare resources [14], and premature mortality [3, 15], which makes multimorbidity one of the major challenges for healthcare systems worldwide [16]. Also, individuals diagnosed with multiple chronic conditions are at an increased risk of premature mortality, hospitalization, and extended hospital stays when compared to those with a single chronic condition [2].

Despite a high prevalence of chronic disease, including diabetes (60%), hypertension (68%), pulmonary disease (45%), and cardiovascular disease (82%) among older adults in Iran, research to identify and characterize determinants of multimorbidity among older population is limited [7, 10, 17, 18]. This study aimed to characterize multimorbidity phenotypes using latent class analysis (LCA): an unbiased, hypothesis-free, model-based method that identifies latent subgroups based on indicator variables within populations with shared characteristics [19]. The use of LCA can prove advantageous in characterizing the clustering of individuals based on the prevalence of chronic conditions while also highlighting the primary disparities between these groups in terms of sociodemographic factors, functioning, and clinical characteristics [20]. Furthermore, by identifying prevalent groups of chronic diseases, policymakers and healthcare professionals may streamline the treatment process for patients with multiple health conditions, and gain a deeper comprehension of the underlying causes of poor health in specific patient demographics [16]. Hence, for the first time in the southern region of Iran, the objective of our study was to examine the correlation between a comprehensive range of sociodemographic factors such as age, gender, smoking, education, income, physical activity, and polypharmacy among individuals aged 60 years or more of the Bushehr province, Iran, by using the LCA clustering. By focusing on Bushehr, a region experiencing rapid aging, our research not only contributes to the broader understanding of multimorbidity but also provides locally tailored insights, essential for informing healthcare strategies and policies in this distinct demographic context. Our aim was to identify the significant factors that contribute to the health outcomes of older adults in our population.

## Materials and methods

### Study design and participants

**Fig. 1:**
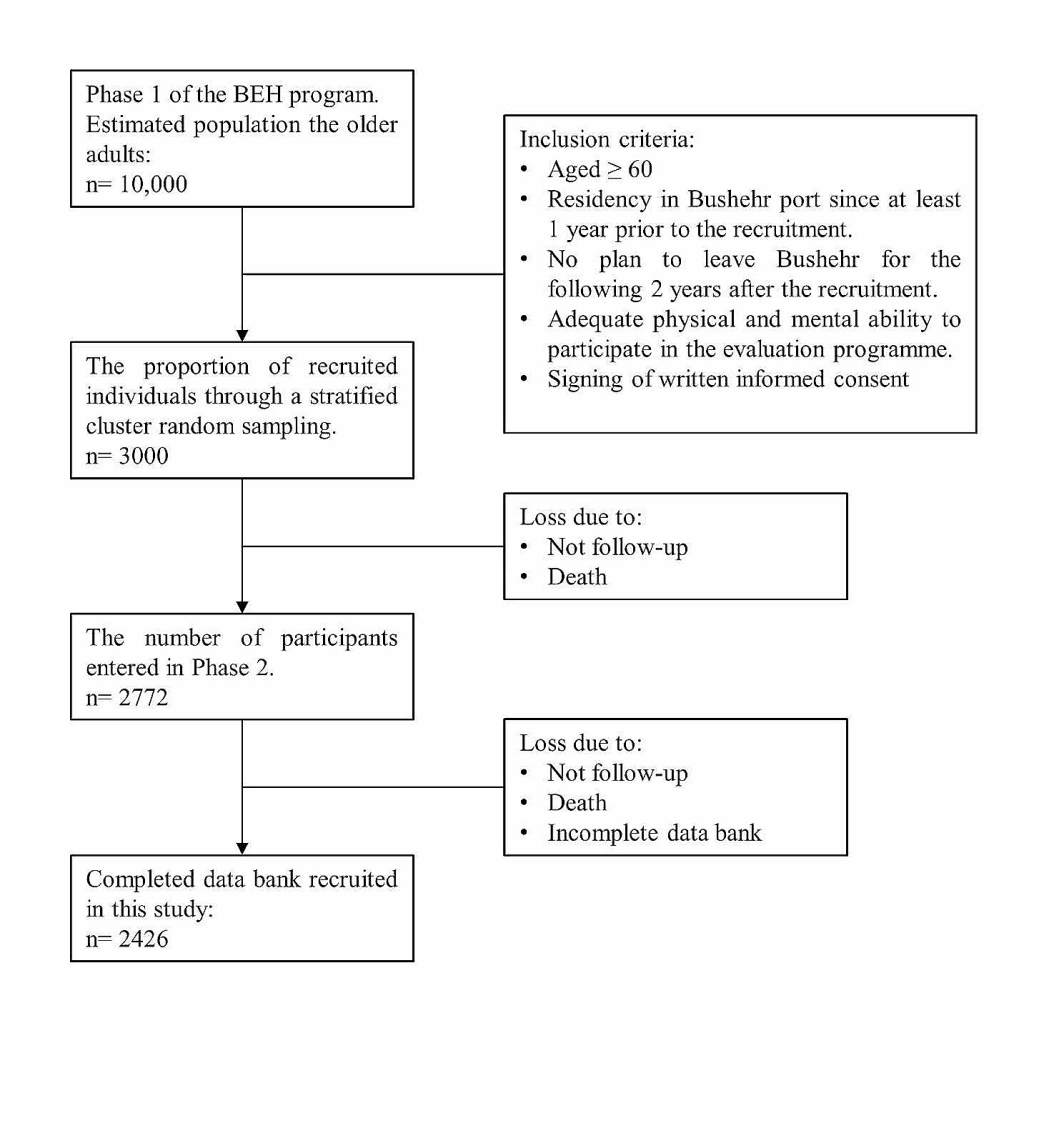
Flow chart of enrolment in the Bushehr Elderly Health (BEH) program

This research was conducted as a secondary cross-sectional analysis on the second phase of the first stage of the Bushehr Elderly Health (BEH) program databank. The BEH program is a prospective cohort study designed to investigate the risk factors associated with non-communicable diseases in individuals aged 60 years and older, residing in Bushehr province, Iran [21, 22]. The participants for this study were chosen using a stratified cluster random sampling technique from an estimated population of 10,000 individuals (as per information obtained from Bushehr health centers). They were invited through various channels, including social media, television, radio, and newspapers, in order to maximize recruitment and reach a diverse range of individuals. Among those who met the criteria, 3,000 individuals accepted to participate in the first stage of the study (Fig. 1). The first stage was enrolled between March 2013 and October 2014. The second stage began in October 2015, intending to estimate the prevalence of musculoskeletal problems, cognitive disorders, age-related chronic diseases including DM, hypertension, stroke, osteoarthritis, depression, thyroid disease (hypothyroid and hyperthyroid), rheumatoid arthritis, obesity, cognitive disorders, osteoporosis and, kidney stone, and the clinical outcomes of participants who had completed the first stage of follow-up. To be eligible for the BEH Program study, certain requirements had to be met by the participants. These requirements included expressing a willingness to be part of the research, being 60 years old or older, having lived in Bushehr for at least a year before the study began, and planning to stay in the city for two years following their involvement in the investigation. In the event that a participant could not provide informed written consent, they were either excluded from the study or their legal guardian provided consent. Comprehensive protocols regarding of the study design of BEH program study, variables, measurement methods, and expected outcomes have been published previously [21, 22]. This current study included 2426 individuals who had completed databank of the required data (including the demographic status, general health, mental and functional health, lifestyle, and medical history).

### Data collection

Trained nursing staff conduct interviews with study participants to gather information through a comprehensive 162-item questionnaire, covering areas such as sociodemographic data, lifestyle factors, general health and medical history, mental and functional health, and medication use [21, 22].

### Sociodemographic variables

These variables included age and sex (confirmed by participant identification card); level of education (illiterate, primary school, high school or higher education); income (low (< 5000 K Iranian Rials), moderate (≥ 5000 K and ≤ 10,000 K Iranian Rials), high (≥ 10,000 K Iranian Rials)) were collected through an interview. The intensity of physical activity in daily work, sports, and free time was calculated using Metabolic Equivalents (METs), with the self-report questionnaire translated into the Farsi language. This tool has been validated for use in older Iranian adults [23, 24]. METs is a measure of the energy expenditure associated with various physical activities. One MET is defined as the energy expended at rest, which is equivalent to the amount of oxygen consumed while sitting quietly. It provides a standardized way to express the energy cost of different activities, allowing for comparisons between them [25]. The degree of physical activity was categorized into four groups: sedentary: 1–1.39; low active: 1.4–1.59; active: 1.6–1.89; and highly active: 1.9–2.5 [26]. Smoking was defined as smoking at least one cigarette per day or smoking hookah at the baseline of the study. Sleep duration was assessed as self-report statements of the mean sleep duration per day through interviews.

### Laboratory evaluations

After a period of fasting that lasted between 8 and 10 h, a qualified nurse drew 25 milliliters of venous blood samples and collected it in labeled containers for further examination. The laboratory tests contained measuring fasting blood sugar (FBS) (mmol/L) and blood lipid profile, which includes total cholesterol, HDL-c, LDL-c, and triglycerides (mmol/L). All of the procedures were carried out by skilled professionals using advanced equipment. Laboratory tests for FBS and blood lipid profile were performed using an enzymatic colorimetric method that utilized a Pars Azmun kit (Pars Azmun, Karaj, Iran).

### Blood pressure assessment

Two measurements of systolic blood pressure (SBP) and diastolic blood pressure (DBP) were taken from the right arm of the participant using a standard mercury sphygmomanometer. The measurements were taken after a 15-minute rest in a seated position, with a time interval of 10 min between them. The average of the two measurements was used to determine the participant’s blood pressure.

### Bone density evaluation

Dual X-ray absorptiometry (DXA) was used to determine the bone density (DXA, Discovery WI, Hologic Inc, USA). Bone mineral density was assessed using a calculation of the T-score in the lumbar spine, neck of the femur, and hip.

### Anthropometric assessments

The measurement of anthropometric indexes followed the standard procedure, which required wearing light clothing and taking off shoes. Height and weight were measured using a fixed stadiometer and a digital scale. Body mass index (BMI) was calculated by dividing weight (in kilograms) by height squared (in meters).

### Measurement of non-communicable diseases and their risk factors

Data for the following 10 non-communicable diseases and risk factors were available in the BEH program and included within the study, with detailed definitions of each provided here: hypertension, diabetes mellitus), stroke, osteoarthritis, depression, thyroid disease, kidney stone, impaired cognition, obesity, and osteoporosis. Hypertension was defined as systolic blood pressure ≥ 140 mmHg, diastolic blood pressure ≥ 90 mmHg, or medication use for hypertension [27]. Diabetes mellitus was defined as an HbA1C ≥ 6.5% (48 mmol/mol), fasting plasma glucose ≥ 126 mg/dl (7.0 mmol/L), or medication use for diabetes [28]. Stroke was defined as a permanent change in sensory, motor activity, or other underlying conditions related to cerebrovascular disease [29]. Based on the previous radiologic findings in the medical records history of individuals or self-reports, the existence of osteoarthritis has been detected. Depression was defined based on physician diagnosis. Thyroid disease was described as a history of thyroid disease requiring medication or otherwise confirmed by a physician. Hypothyroidism was defined as a thyroid-stimulating hormone (TSH) over 10 mIU/L, and hyperthyroidism was defined as TSH below 0.4 mIU/L. The history of kidney stones was attributed to having renal stones or positive history of renal passage.

The categorical verbal fluency test (CFT) was used to assess cognitive function and categorized participants as having normal or impaired cognition. CFT is a neuropsychological assessment tool used to evaluate an individual’s cognitive function, specifically their verbal fluency. Verbal fluency refers to the ability to generate words or phrases quickly and efficiently within a specified category or set of rules [30]. In the CFT, the cut-point for participants with an education level less than primary school was 12, and the cut-point for participants with a level of education higher than primary school was 14. Obesity was defined as a body mass index (BMI) of 30.0 kg/m^2^ or greater. For assessing osteoporosis, a T-score higher than − 1 was considered normal, while scores below − 2.5 and between − 2.5 to -1 were considered osteoporosis and osteopenia, respectively [31]. Polypharmacy was defined as the use of five or more medications [32] (as reported during the survey questionnaire).

### Consent and ethics

This study was conducted in full accordance with the Declaration of Helsinki. Ethical approval for this study was granted by the Ethics Committee of Bushehr University of Medical Sciences (IR.BPUMS.REC.1402.062). All study participants provided informed consent to participate in this study, after learning about the procedures involved. Participation was entirely voluntary; any participant was able to withdraw their consent at any time, without consequence.

### Risk of bias

In this study, we took several measures to minimize bias and ensure accurate data collection. Firstly, we used validated and standard questionnaires to collect data. Secondly, we employed trained and professional interviewers to remind individuals or their guardians of all outcome aspects and reduce the risk of recall bias. Thirdly, we used a stratified cluster random sampling technique to ensure that the study participants were as representative of their population as possible. Moreover, if applicable, we cross-checked the data collected through questionnaires and interviews against objective measurements such as laboratory data, medical records, and physical and anthropometric evaluations to confirm their accuracy.

### Statistical analysis

The study population was described using counts and percentages for categorical variables, means and standard deviations for normally distributed continuous variables, and medians and interquartile ranges for non-normally distributed variables. Differences between males and females were tested using Chi-squared tests, and those between normally-distributed continuous variables were evaluated using *t*-tests. Latent class analysis (LCA) was performed to elucidate discrete multimorbidity classes, which depict complex patterns of higher order interactions between the 10 long-term health conditions and risk factors outlined above. The LCA model was fitted with between zero and seven classes. Long-term diseases present in the study cohort with a prevalence of 3% or greater were included in the LCA model (10 disease states in total), as in previous studies [9, 33, 34]. The optimal class solution was chosen based on a compromise between maximizing clinical interpretability and model parsimony, whilst minimizing the likelihood-ratio statistic, G^2^, the Akaike Information Criterion (AIC), and the Bayesian Information Criterion (BIC). An elbow plot was used to identify the point at which the log-likelihood started to level off.

After selecting the optimal model, multinomial logistic regression was performed to identify the effect of covariates on latent class membership. These covariates were age, gender, education, household income, marital status, physical activity, smoking status, and polypharmacy. Missing data were negligible (< 2% for each variable), as such we anticipated minimal impact from these missing data on the analyses and conducted a complete case analysis.

Statistical analyses were performed using Stata 14 (population characteristics, and multinomial logistic regression analysis) and R version 4.1.1 (LCA). In all analyses, a p-value below the threshold of 0.05 was considered statistically significant.

## Results

A total of 2,419 participants over the age of 60 were included in this analysis. The mean age of participants was 69 ± 6 years. The majority of subjects were women (51.9%), 67% and 76.8% of our study population were literate and married, respectively. The overall prevalence of multimorbidity (≥ 2 chronic diseases) was 80.2% (*n* = 1946). Table 1 shows the prevalence of long-term conditions with a prevalence of > 3% in our sample, stratified by sex. The three most common disease states were hypertension (73%), cognitive disorders (49.2%), and osteoporosis (41.4%).

**Table 1:** Study characteristics, stratified by gender

Characteristics	Categories	Total (*n* = 2426)	Male (*n* = 1161)	Female (*n* = 1265)	P-value
		No. (%)	No. (%)	No. (%)	
Age (mean ± SD)		69.3 ± 6.3	69.5 ± 6.4	69.1 ± 6.3	0.165
Marital status	Married	1864(76.8)	1104(95.1)	760(60.1)	< 0.001
	Single	562(23.2)	57(4.9)	505(39.9)	
Educational level	Illiterate	801(33.0)	214(18.4)	587(46.4)	< 0.001
	Primary school	712(29.3)	324(27.9)	388(30.7)	
	High school diploma	718(29.6)	466(40.1)	252(19.9)	
	Academic	195(8.0)	157(13.5)	38(3.0)	
Physical Activity	No active and sedentary	1869(77.0)	893(76.9)	976(77.2)	0.087
	Low active	399(16.4)	180(15.5)	219(17.3)	
	Active	158(6.5)	88(7.6)	70(5.5)	
Household Income	Low	513(21.1)	168(14.5)	345(27.3)	< 0.001
	Moderate	1362(56.1)	641(55.2)	721(57.0)	
	High	551(22.7)	352(30.3)	199(15.7)	
Tobacco smoking	No	737(30.4)	321(27.6)	416(32.9)	0.001
	Past smoking	1185(48.8)	568(48.9)	617(48.8)	
	Current smoking	504(20.8)	272(23.4)	232(18.3)	
Sleep	Less than 6 h	46(1.9)	24(2.1)	22(1.7)	0.832
	6 to 9 h	1682(69.3)	805(69.3)	877(69.3)	
	More than 9 h	698(28.8)	332(28.6)	366(28.9)	
Polypharmacy		339(17.0)	88(9.8)	251(22.8)	< 0.001
Hypertension		1770(73.0)	819(70.5)	951(75.2)	0.010
History of Stroke		81(3.3)	37(3.2)	44(3.5)	0.735
History of Osteoarthritis		305(12.6)	95(8.2)	210(16.6)	< 0.001
History of Depression		623(25.7)	154(13.3)	469(37.1)	< 0.001
History of Diabetes mellitus		767(31.6)	327(28.2)	440(34.8)	< 0.001
History of Thyroid disease		231(9.5)	44(3.8)	187(14.8)	< 0.001
History of Obesity		645(26.6)	188(16.2)	457(36.1)	< 0.001
History of Cognitive disorder		1194(49.2)	451(38.8)	743(58.7)	< 0.001
History of Osteoporosis		1005(41.4)	275(23.7)	730(57.7)	< 0.001
History of Kidney stones		212(8.7)	118(10.2)	94(7.4)	0.018
Multi-morbidity (2 + chronic conditions)		1946(80.2)	794(68.4)	1152(91.1)	< 0.001
Number of co-occurring diseases (mean ± SD)		2.8 ± 1.4	2.1 ± 1.2	3.4 ± 1.4	< 0.001

### Latent class analysis (LCA) results

**Fig. 2:**
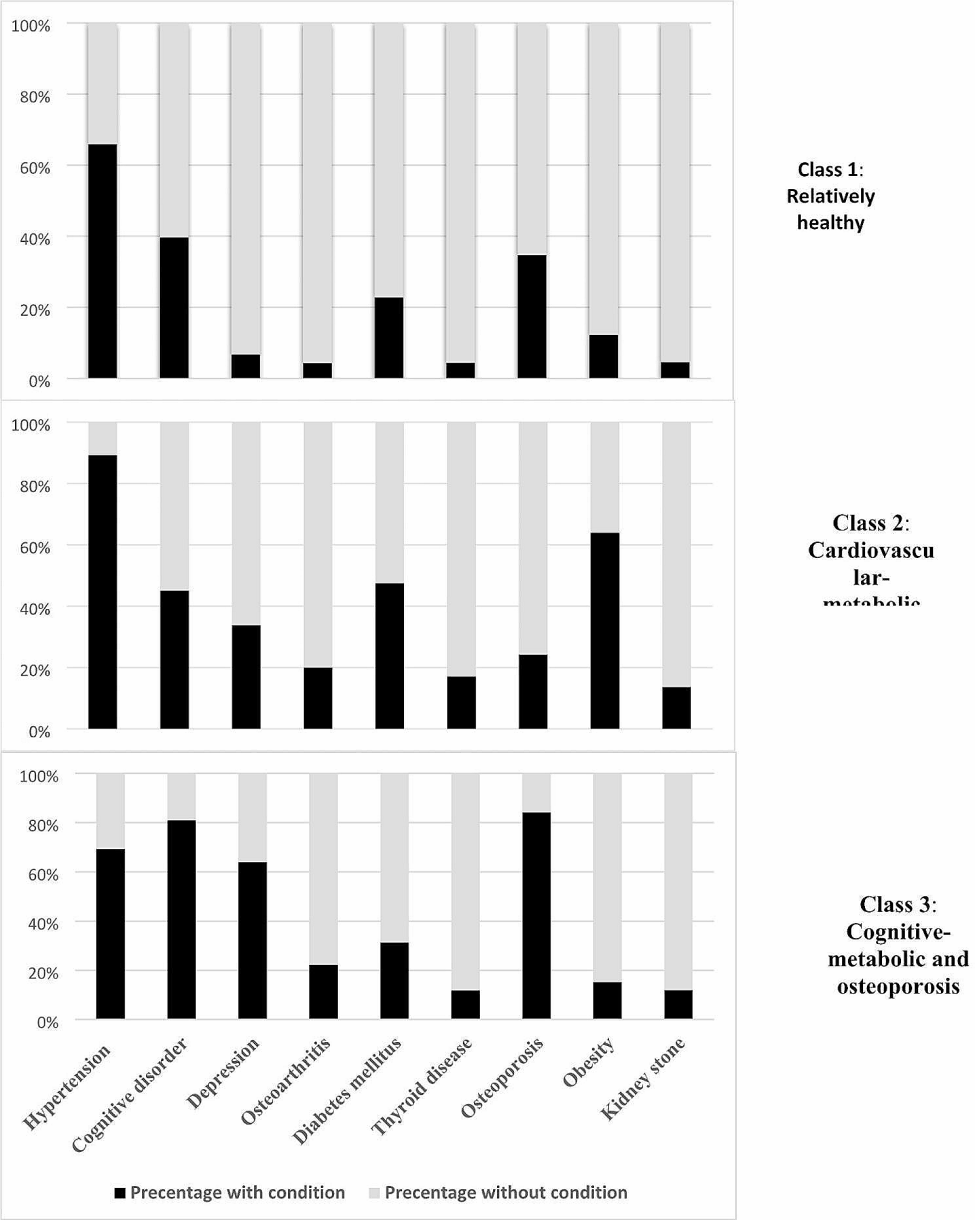
Item-response probabilities of multimorbid conditions within each multimorbidity cluster. Participants were categorized as Class 1 (Relatively healthy), Class 2 (Cardiovascular-metabolic), and Class 3 (Cognitive-metabolic and osteoporosis)

The results of LCA models including from 1 to 7 classes are presented in Table 2. The three-class model was determined to offer the best compromise between clinical interpretability, model parsimony, and model fit. The item-response probabilities of multimorbid conditions within each multimorbidity cluster are illustrated in Fig. 2.

**Table 2:** Comparison of latent class analysis models with different numbers of latent classes based on model selection statistics

Number of latent class	Number of parameters estimated	G^2^	df	AIC	BIC	X^2^	Entropy	Maximum log-likelihood
1	9	953	502	22,904	22,956	1584	4.71	-11443.10
2	19	691	492	22,661	22,771	857	4.66	-11311.95
3	**29**	**510**	**482**	**22,501**	**22,669**	**668**	**4.63**	**-11221.81**
4	39	457	472	22,468	22,694	575	4.61	-11195.19
5	49	425	462	22,456	22,740	528	4.61	-11179.19
6	59	389	452	22,440	22,782	507	4.60	-11161.22
7	69	366	442	22,437	22,837	491	4.59	-11149.83

*Note* LCA: latent class analysis; AIC: Akaike information criterion; BIC: Bayesian information criterion. df: degree of freedom; G^2^: the likelihood-ratio statistic

Table 3 shows the distribution of long-term health conditions among each distinct class of the three-class model, among those participants with multimorbidity. The classes were assigned names based on the results of this analysis. Class 1 has the highest prevalence among the study sample (56.9%), with no clear emergent inter-dependent patterns of individual comorbidities. As such, we have labeled this the ‘relatively healthy’ class. Class 2 has an overall prevalence of 25.8%, and contains a high prevalence of individuals with hypertension, diabetes, and obesity, and as such, we have labeled this the cardiovascular-metabolic class. Class 3 has a lower overall prevalence in the cohort (17.1%), but is enriched with a high burden of hypertension, cognitive impairment, depression, and osteoporosis. As such, we have labeled this the cognitive-metabolic and osteoporosis class.

**Table 3:** Class membership and item response probabilities of the three latent classes

Latent class			
	Relatively healthy (1)	Cardiovascular-metabolic (2)	Cognitive-metabolic and osteoporosis (3)
Latent class prevalence	0.574(1394)	0.256(623)	0.168(409)
Item-response probabilities			
Hypertension	**0.659**	**0.893**	**0.694**
Cognitive impairment	0.396	0.451	**0.810**
Osteoarthritis	0.043	0.200	0.223
Diabetes mellitus	0.228	**0.476**	0.313
Thyroid disease	0.044	0.170	0.118
Obesity	0.122	**0.639**	0.152
Depression	0.067	0.339	**0.639**
Osteoporosis	0.347	0.243	**0.842**
Kidney stone	0.046	0.137	0.120

*Note* The probability of a “No” response can be calculated by subtracting the item-response probabilities shown above from 1

^*^Item-response probabilities > 0.5 in bold to facilitate interpretation

The sociodemographic characteristics of the study population, stratified by latent class membership, are presented in Table 4. The mean age was different between the three classes, with members of the cognitive-metabolic and osteoporosis class being older on average (71.2 years, vs. 69.4 in the relatively healthy group, *p* < 0.001). Class 3 (cognitive-metabolic and osteoporosis) had a higher membership of women (79.9%) and the highest overall number of diagnoses on average (4.2). Members of this class tended to be less educated and more sedentary than the other classes. The membership of class 1 (relatively healthy) was predominantly male (61.0%) and tended to have greater household income when compared with the other classes.

**Table 4:** Characteristics of study participants, stratified by latent class membership

Characteristics	Categories	Class 1: Relatively healthy (*n* = 1394)	Class 2: Cardiovascular-metabolic (*n* = 623)	Class 3: Cognitive-metabolic and osteoporosis (*n* = 409)	P-value^*^
		No. (%)	No. (%)	No. (%)	
Age (Mean ± SD) ^*^		69.4 ± 6.4	67.9 ± 5.1	71.2 ± 7.4	< 0.001
Number of co-occurring diseases*		1.9 ± 1.0	3.8 ± 1.2	4.2 ± 1.0	< 0.001
Gender**	Male	847(60.8)	230(36.9)	84(20.5)	< 0.001
	Female	547(39.2)	393(63.1)	325(79.5)	
Marital status**	Married	1138(81.6)	476(76.4)	250(61.1)	< 0.001
	Single	256(18.4)	147(23.6)	159(38.9)	
Educational level**	Illiterate	379(27.2)	198(31.8)	224(54.8)	< 0.001
	Primary school	409(29.3)	188(30.2)	115(28.1)	
	High school Diploma	465(33.4)	195(31.3)	58(14.2)	
	Academic	141(10.1)	42(6.7)	12(2.9)	
Physical Activity**	No active and sedentary	1040(74.6)	492(79.0)	337(82.4)	0.005
	low active	246(17.6)	97(15.6)	56(13.7)	
	Active	108(7.7)	34(5.5)	16(3.9)	
Household Income**	Low	252(18.1)	123(19.7)	138(33.7)	< 0.001
	Moderate	778(55.8)	370(59.4)	214(52.3)	
	High	364(26.1)	130(20.9)	57(13.9)	
Tobacco smoking**	No	425(30.5)	204(32.7)	108(26.4)	0.002
	Past smoking	662(47.5)	322(51.7)	201(49.1)	
	Current smoking	307(22.0)	97(15.6)	100(24.4)	
Sleep**	Less than 6 h	20(1.4)	12(1.9)	14(3.4)	< 0.001
	6 to 9 h	1024(73.5)	433(69.5)	225(55.0)	
	More than 9 h	350(25.1)	178(28.6)	170(41.6)	
Polypharmacy**	Yes	98(9.3)	171(29.4)	70(19.7)	< 0.001
	No	958(90.7)	411(70.6)	285(80.3)	

*ANOVA

**Chi-square

### Multinomial logistic regression results

Multinomial logistic regression was performed to identify associations between sociodemographic factors and each multimorbidity class (Table 5). Briefly, older age reduced the odds of membership in cardiovascular-metabolic class (odds ratio [OR] 0.95, 95% confidence intervals [CI] 0.93–0.97) class compared to relatively healthy class. The odds of membership of cognitive-metabolic and osteoporosis class (OR 4.45, 95% CI 3.17–6.25) and cardiovascular-metabolic class (OR 1.98, 95% CI 1.53–2.55) were significantly higher for women than men, compared to relatively healthy class. Those with greater education levels were less likely to belong to cognitive-metabolic and osteoporosis class, compared with relatively healthy class (OR 0.43, 95% CI 0.29–0.64). Smoking was associated with a greater risk of membership in cognitive-metabolic and osteoporosis class, compared with relatively healthy class (OR 1.46, 95% CI 1.01–2.12). Those with a low physical activity was less likely to be a member of cardiovascular-metabolic class than those with no active (OR = 0.74, 95% CI: 0.55–0.99). Polypharmacy was associated with membership in cardiovascular-metabolic class (OR = 3.52, 95% CI: 2.65–4.68) and cognitive-metabolic and osteoporosis class (OR = 1.84, 95% CI: 1.28–2.63) compared to relatively healthy class. Having enough sleep (6 to 9 h) was associated with a lower likelihood of being a member of cognitive-metabolic and osteoporosis class (OR 0.18, 95% CI 0.07–0.44).

**Table 5:** Summary findings of multinomial logistic regression of association between multimorbidity and some of its risk factors

Variables	Categories	Class1: Relatively healthy(*n* = 1394) OR(95%CI)	Class 2: Cardiovascular-metabolic(*n* = 623) OR(95%CI)	Class3: Cognitive-metabolic and osteoporosis(409) OR(95%CI)
Age		1	0.95(0.93–0.97)*	1.01(0.99–1.03)
Gender	♦ Male	**1**	**1**	**1**
	Female	1	1.98(1.53–2.55)*	4.45(3.17–6.25)*
Marital status	♦Married	**1**	**1**	**1**
	Single	1	0.81(0.68–1.27)	0.84(0.61–1.16)
Educational level	♦ Illiterate	**1**	**1**	**1**
	Primary school	1	0.95(0.71–1.26)	0.69(0.50–0.94)*
	High school Diploma	1	0.88(0.69–1.27)	0.43(0.29–0.64)*
	Academic	1	0.79(0.53–1.37)	0.52 (0.25–1.05)
Physical Activity	♦No active and sedentary	**1**	**1**	**1**
	low active	1	0.74(0.55–0.99)*	0.74(0.51–1.09)
	Active	1	0.64(0.40–1.01)	0.66(0.36–1.23)
Household Income	♦Low	1	**1**	**1**
	Moderate	1	0.96(0.72–1.29)	0.79(0.58–1.08)
	High	1	0.72(0.50–1.04)	0.70(0.45–1.09)
Tobacco smoking	♦No	1	**1**	**1**
	Past smoking	1	1.04 (0.81–1.32)	1.02(0.75–1.39)
	Current smoking	1	0.80(0.58–1.11)	1.46(1.01–2.12)*
Sleep	♦Less than 6 h	**1**	**1**	**1**
	6 to 9 h	1	0.50(0.21–1.18)	0.18(0.07–0.44)*
	More than 9 h	1	0.65(0.27–1.53)	0.34(0.13–0.83)*
Polypharmacy	♦No	**1**	**1**	**1**
	Yes	1	3.53(2.65–4.69)*	1.96(1.37–2.80)*

*P-value < 0.05; OR: odds ratio; CI: confidence interval. Class 1 (background ageing) was considered the reference group for multinomial regression analyses. ♦ indicates the reference category for each covariate

## Discussion

Despite projections suggesting a marked increase in the number of older adults living in low- and middle-income countries in the coming decades, there has been little research into the epidemiology of multimorbidity in this setting to date [7]. This study is the first to investigate the prevalence of multimorbidity and to characterize determinants of three latent multimorbidity phenotypes identified among a community-dwelling older adult population in the Bushehr Elderly Health program in Iran. The Latent Multimorbidity Classes approach is a method that is used to identify subgroups within a population that have co-existing medical conditions. This approach categorizes individuals based on their specific patterns of chronic health conditions and provides insights into diverse subgroups within a population. The information gained through this method can be helpful in developing tailored healthcare strategies for managing multiple morbidities. We identified three distinct latent multimorbidity classes in our study population using an *a priori* hypothesis-free approach, which putatively represented background aging (class 1), predominant cognitive-metabolic multimorbidity (class 2), and cardiovascular-metabolic multimorbidity (class 3).

Based on our results, over two-thirds of participants had multimorbidity, which was higher than in previous studies. The prevalence of multimorbidity we observed in this study of older adults in Iran (80.2%) was markedly higher than that previously reported in Germany, the United Kingdom, and Qatar (40%, 27.2%, and 22%, respectively( [35–37]. The prevalence of multimorbidity in this study was even higher in comparison with that reported in other low- and middle-income countries, including Vietnam (63%) and China (34.7%) [3, 4]. In our study, the simultaneous occurrence of DM, hypertension, and obesity could be a precursor to the onset of metabolic disorders and increase the risk of cardiovascular disease. In Iran, hypertension, DM, musculoskeletal conditions, fatty liver, and heart diseases were the most common chronic diseases [10].

While increased age is expected to lead to a higher risk of multimorbidity, it was observed in our results that higher age was associated with a lower risk of cardio-metabolic disorders. The explanation for this unexpected finding and outwith common knowledge of cardiovascular risk [9, 38, 39] might be due to the narrow age profile of our study population (more than 70%of the population was between 65 and 70 years old). In addition, it may be possible that we are observing a survivorship bias in that those people with the worst cardio-metabolic disease die young so that, by definition, the oldest surviving patients with the cardio-metabolic disease have less severe disease.

Our study identified three distinct multimorbidity classes in a contemporary Iranian population of older adults, representing background aging, cognitive-metabolic disease, and cardiovascular-metabolic disease. This study highlights the association of female gender and polypharmacy with cognitive-metabolic and cardiovascular-metabolic disease classes. Also, people with current smoking consumption were more prone to have a cognitive metabolic class of multimorbidity. Moreover, having an active lifestyle and a higher age were determined as protective factors against cardiovascular metabolic multimorbidity; sleep ≥ 6 h a day, and a higher level of education indicated as protective factors for the occurrence of multimorbidity.

According to the present study, being female significantly increased the odds of membership in the cognitive-metabolic and cardiovascular-metabolic classes. These results may be attributed to a complex interplay of multiple factors, including biological differences, hormonal influences, gender-specific lifestyle and behavioral factors, socioeconomic disparities, genetic predispositions, and healthcare-seeking behavior. This finding concurs with previous studies in which multimorbidity has been shown to be higher in females than males [13, 40]. Several reasons are thought to contribute to this difference, including insulin resistance and increased triglyceride levels, systolic and diastolic blood pressure [41] due to higher waist circumference among women, estrogen levels which contribute to the risk of depression [42], and menopause contributing to cardiovascular risk [43] as well as a generally longer life expectancy among women [17]. According to the available evidence, there is a significant disparity between men and women in disease co-occurrence due to long-term variations in risk factors related to multimorbidity, such as lipid profile, body mass index, estrogen deficiency, and menopause. Given the unequal distribution of healthy life expectancy between genders, it appears necessary to implement appropriate healthcare interventions for older adult women [9, 44].

Polypharmacy was also strongly associated with two classes of multimorbidity in our study. It was associated with the odds of the cardiovascular-metabolic class by approximately 3.5-fold and the odds of the cognitive-metabolic class by 1.84-fold. Three main consequences have been reported for polypharmacy: (i) adverse drug reactions (ADR), (ii) risk of falls, and (iii) compliance. ADR consists of the direct effect of one drug, or the interaction between different drugs and can be associated with increased morbidity and mortality. Polypharmacy is also associated with an increased falls risk, which can lead to hip fractures and disability. Drug classes that are particularly associated with falls risk include diuretics, benzodiazepine derivatives, and anticholinergic agents. Taking more drugs per day decreases compliance and adherence to prescriptions resulting in poor effectiveness of drugs [45]. Finally, polypharmacy may cause appetite loss, nausea, diarrhea, weight change, taste impairment, declined saliva secretion, change in lipid profile, dysregulation in electrolytes balance, and altered glucose metabolism, which leads to worsening the nutritional status in the elderly [46]. It seems that older age is associated with the risk of multimorbidity and polypharmacy, and the simultaneous occurrence of these two factors is associated with progressive loss of resilience and impaired homeostasis. As a result, it can lead to an increased burden on health and social care. Therefore, healthcare professionals must prioritize appropriate assessments of frailty and medication optimization when caring for older patients. Such measures can improve patient outcomes and reduce the burden on healthcare systems [47].

The present study showed that increased physical activity lowered the chance of having cardiovascular-metabolic class multimorbidity, and increased education level reduced the odds of membership in the cognitive-metabolic class. These findings emphasize the importance of lifestyle choices and education in influencing health. Clustering cardiometabolic risk factors (such as visceral obesity, hypertension, hyperglycemia, and dyslipidemia) in an individual leads to additional susceptibility risk for cardiometabolic diseases, diabetes mellitus type 2, and all-cause mortality. It has been shown that declined moderate-to-vigorous physical activity is a significant risk factor for clustered cardio-metabolic disease, and having an active lifestyle can improve waist circumference and HDL-c level [48]. Based on a meta-analysis of 36 studies including more than 3 million participants observed for a median period of 12 years, physical activity related to a 17% reduced risk of cardiovascular events, a 23% decreased risk of cardiovascular mortality, and a 26% lower incidence of diabetes [49]. According to this evidence, individuals with multimorbidity, particularly cardiometabolic diseases, report lower levels of physical activity. Therefore, promoting targeted physical activity strategies in these people can improve their quality of life due to the systematic role of exercise in protecting the heart, reducing oxidative stress and chronic inflammation, promoting stem cell mobilization, and improving cardiovascular health [50]. In previous reports also, it has been mentioned that higher level education was related to better cognitive function, and people with dementia had lower education levels [51]. Higher levels of education might enable people to perform a prolonged level of higher cognitive function and delay the onset of deterioration [52]. It seems that the occurrence of multimorbidity is concentrated in people with low education. Therefore, education level as one of the determinants of health has a direct effect on healthy and unhealthy lifestyles, living conditions, levels of stress and pressures, social disadvantages during life, and suffering from chronic diseases [9, 53].

In addition, current smoking status was associated with an increased risk of cognitive multimorbidity class in our study. Likewise, the damaging effects of tobacco use on physical and mental health have been reported previously [54, 55]. Boksa believes that smoking is associated with changes in the brain’s structure and neural circuitry in brain systems, which can cause many psychiatric disorders. It can also modulate the effects of psychotropic drugs. Thus, smoking can impact the process of mental illnesses and brain function [56]. Our results demonstrated a relationship between sleep duration and the cluster of cognitive-metabolic multimorbidity, as it would be the chance of having this class of multimorbidity was 83% lower in individuals with sleeping 6 to 9 h per day, compared with those who slept < 6 h. A pooled cohort study also showed an inverted U-shaped association between sleep duration and cognitive function. They noted that sleeping < 4 or > 10 h per night contributed to lower cognitive function at baseline and its faster decline during the follow-ups. A possible explanation for that could be the thinning of the cerebral cortex due to sleeping more or less than 7 h. The other reasons could be the dysregulation of interleukin-6 and C-reactive protein inflammatory pathways due to excessive sleep duration, increased hippocampal synaptic plasticity because of short periods of sleep deprivation, and deposition of amyloid plaques as the etiological factor of Alzheimer’s disease [57]. Other studies also reported the inverted U-shaped relationship [58, 59]. According to a cohort study, short sleep duration and poor sleep quality are linked to an increased risk of developing multimorbidity in elderly people. Therefore, it is important to consider optimal sleep duration and quality to prevent and control multimorbidity [60].

Finally, LCA differs from other statistical methods used in cohort studies in that it is a person-centered mixed model analysis that identifies similar subgroups or clusters of individuals in a population that may benefit from targeted interventions or treatments, while other statistical methods may not provide this accuracy. This approach can provide insight into the population structure that may not be possible with other statistical methods [19]. In addition, unlike other clustering methods, it allows objective testing of model fit [61].

Our study suggests that potential interventions could be developed based on specific multimorbidity classes identified through latent class analysis, which could enhance precision in healthcare. To achieve this, exploring targeted strategies for each subgroup is necessary. Also, exploring intervention and health strategies to reduce associated factors within each latent class is crucial in addressing the unique health challenges of each subgroup. Furthermore, the versatility of latent class analysis in various research populations with different specifics could be further explored, extending its application beyond what we have done in our current study. This will pave the way for gaining deeper insights into multimorbidity across different populations and healthcare scenarios.

## Strengths and limitations

This study was conducted in a large, comprehensively phenotyped population of older Iranian adults, using a validated protocol and advanced modeling methods. However, this study had several potential limitations. Firstly, we were limited to considering only specific chronic disease conditions recorded in the program and could not consider all possible chronic diagnoses. We looked at diseases with a more than 3% prevalence, as in previous studies [9, 33]. In a sensitivity analysis, the addition of diseases with a prevalence of less than 3% to our model did not change the emerging multimorbidity clusters. Another limitation of this study is its cross-sectional nature, which provides evidence for the association between factors but does not establish the directionality or causality of these associations. Furthermore, it was not possible to assess the effect of the multimorbidity classes on outcomes (such as mortality) using the data available In addition, the self-report method was used to measure height and weight for a limited number of elderly people who could not stand and had skeletal disorders. We believe further multi-region, longitudinal studies with a broader age distribution might help to clarify the relationship between chronic diseases and multimorbidity in the older population, and importantly their impact on long-term outcomes.

## Conclusion

Multimorbidity was common among this sample of older Iranian adults– over two-thirds had two or more long-term health conditions. Diseases such as hypertension, diabetes, obesity, depression, and cognitive impairments all commonly contributed to the multimorbidity state in older adults. Furthermore, being of female gender, education level, tobacco, sleep duration, and polypharmacy were important predictors of being in the cardiovascular-metabolic and cognitive-metabolic and osteoporosis class. So that being female and polypharmacy significantly increased the odds of multimorbidity. By using an a priori hypothesis-free modeling approach, this study identified three common latent multimorbidity phenotypes that can be identified among older Iranian adults with morbidity. These latent multimorbidity classes were associated with different baseline demographic factors. Further work is required to evaluate the impact of these phenotypes on the quality of life and clinical outcomes in this population. Eventually, we hope that applying targeted interventions early in the development of specific multimorbidity phenotypes may help to reduce multimorbidity and lead to improvements in the functional status, quality of life, and lifespan of older adults.

## Data Availability

Upon a reasonable request, the datasets used during the current study are available from the corresponding authors.
